# Effects of herbal cake-partitioned moxibustion on the expression of thyroid autophagy-related factors LC3B and Beclin-1 in rats with autoimmune thyroiditis

**DOI:** 10.1007/s11726-022-1345-1

**Published:** 2022-12-23

**Authors:** Kexu Chen, Jimeng Zhao, Yu Qiao, Lu Zhu, Yan Huang, Yanan Liu, Handan Zheng, Huirong Liu, Yunhua Cui, Huangan Wu

**Affiliations:** 1 Yueyang Hospital of Integrated Traditional Chinese and Western Medicine, Shanghai University of Traditional Chinese Medicine, Shanghai, 200437 China; 2 Shanghai Research Institute of Acupuncture and Meridian, Shanghai, 200030 China

**Keywords:** Moxibustion Therapy, Medicinal Cake-partitioned Moxibustion, Thyroiditis, Autoimmune, Autophagy, Points, Conception Vessel, Points, Governor Vessel, Rats, 灸法, 药饼灸疗法, 甲状腺炎, 自身免疫性, 自噬, 穴位, 任脉, 穴位, 督脉, 大鼠, R2-03, A

## Abstract

**Objective:**

To observe the anti-inflammatory effect, as well as the effect on the expression of microtubule-associated protein light chain 3B (LC3B) and Beclin-1 of herbal cake-partitioned moxibustion in rats with experimental autoimmune thyroiditis (EAT).

**Methods:**

Female Sprague-Dawley rats were randomly divided into a normal group and a modeling group. The EAT rat model was prepared by a combination of antigen immunization plus iodine agent induction. After the model was prepared, rats in the modeling group were randomly and equally divided into a model group and a herbal cake-partitioned moxibustion group. In the herbal cake-partitioned moxibustion group, moxibustion was alternately applied to two groups of points [Dazhui (GV14)-Mingmen (GV4) and Tiantu (CV22)-Guanyuan (CV4)], and the treatment continued for 30 d. Rats in the normal and model groups were only fixed identically without intervention. Histopathological manifestations of thyroid glands were observed by hematoxylin-eosin staining; the concentrations of thyroid peroxidase antibodies (TPOAb), thyroglobulin antibodies (TGAb), interleukin-1β (IL-1β), interleukin-6 (IL-6), and tumor necrosis factor-α (TNF-α) were detected by enzyme-linked immunosorbent assay; real-time fluorescence quantitative polymerase chain reaction and immunohistochemistry were used to detect the mRNA and protein expression of autophagy-related factors LC3B and Beclin-1 in thyroid tissue.

**Results:**

There were massive follicular destruction, lymphocytic infiltration, and interstitial fibrous tissue hyperplasia of the thyroid glands in the model group. Some follicles of the thyroid glands were destroyed with few lymphocyte infiltrations and fibrous tissue hyperplasia in the moxibustion group. Compared with the normal group, the concentrations of serum TPOAb, TGAb, IL-1β, IL-6, and TNF-α were increased in the model rats (*P*<0.05); the mRNA and protein expression levels of LC3B and Beclin-1 in thyroid tissue were reduced in the model group (*P*<0.05). Compared with the model group, the concentrations of serum TPOAb, TGAb, IL-1β, IL-6, and TNF-α were reduced in the herbal cake-partitioned moxibustion group (*P*<0.05); the mRNA and protein expression levels of LC3B and Beclin-1 in thyroid tissue were increased in the herbal cake-partitioned moxibustion group (*P*<0.05). The mRNA and protein expression of LC3B and Beclin-1 in thyroid tissue was negatively correlated with the serum levels of TPOAb and TGAb.

**Conclusion:**

Herbal cake-partitioned moxibustion reduces the inflammatory response in the thyroid glands of EAT rats and lowers the levels of serum TPOAb and TGAb. This may be related to the regulation of mRNA and protein expression of the autophagy-associated factors LC3B and Beclin-1 in rat thyroid tissue.

## Abstract (zh)

**目的:**

观察隔药饼灸干预实验性自身免疫性甲状腺炎(EAT)大鼠的抗炎效应, 以及对自噬相关因子微管相关 蛋白轻链3B(LC3B)及Beclin-1表达的影响。

**方法:**

将雌性Sprague-Dawley大鼠随机分为正常组和造模组, 采用抗原免 疫加碘剂联合诱导的方法制备EAT大鼠模型。模型制备完成后将造模组大鼠随机等分为模型组和隔药饼灸组。隔 药饼灸组在两组穴位(大椎-命门和天突-关元)交替进行隔药饼灸治疗, 连续干预30 d。正常组和模型组只做相同固定, 不进行干预治疗。采用苏木素-伊红染色法观察甲状腺组织病理学表现; 采用酶联免疫吸附剂测定法检测血清抗甲 状腺过氧化物酶抗体(TPOAb)、抗甲状腺球蛋白抗体(TGAb)、白细胞介素-1β(IL-1β)、白细胞介素-6(IL-6)和肿瘤坏死 因子-α(TNF-α)的浓度; 采用实时荧光定量多聚核苷酸链式反应法和免疫组化法检测甲状腺组织自噬相关因子LC3B 和Beclin-1的mRNA及蛋白表达。

**结果:**

模型组甲状腺大量滤泡破坏, 有大量淋巴细胞浸润, 间质大量纤维组织增生; 隔 药饼灸组甲状腺部分滤泡破坏, 有少量淋巴细胞浸润, 少量纤维组织增生。与正常组比较, 模型组大鼠血清TPOAb、 TGAb、IL-1β、IL-6和TNF-α的浓度均升高(*P*<0.05); 模型组甲状腺组织LC3B和Beclin-1的mRNA与蛋白表达水平均降低 (*P*<0.05)。与模型组相比, 隔药饼灸组大鼠血清TPOAb、TGAb、IL-1β、IL-6和TNF-α的浓度均降低(*P*<0.05); 隔药饼灸组 甲状腺组织LC3B和Beclin-1的mRNA与蛋白表达水平均升高(*P*<0.05)。甲状腺组织LC3B和Beclin-1的mRNA及蛋白表达 水平与血清TPOAb及TGAb的浓度均呈负相关。

**结论:**

隔药饼灸能减轻EAT大鼠甲状腺的炎性反应, 降低血清TPOAb及 TGAb水平。这可能与调节大鼠甲状腺组织自噬相关因子LC3B和Beclin-1的mRNA及蛋白表达有关。

**First Author**: CHEN Kexu, master degree candidate
**Joint Corresponding Authors**: WU Huangan, M.D., professor, doctoral supervisor. E-mail: wuhuangan@126.com; CUI Yunhua, M.D., associate researcher, master student supervisor. E-mail: cui_yunhua@126.com

药饼灸组在两组穴位(大椎-命门和天突-关元)交替进行隔药饼灸治疗, 连续干预30 d。正常组和模型组只做相同固定, 不进行干预治疗。采用苏木素-伊红染色法观察甲状腺组织病理学表现; 采用酶联免疫吸附剂测定法检测血清抗甲状腺过氧化物酶抗体(TPOAb)、抗甲状腺球蛋白抗体(TGAb)、白细胞介素-1β(IL-1β)、白细胞介素-6(IL-6)和肿瘤坏死因子-α(TNF-α)的浓度; 采用实时荧光定量多聚核苷酸链式反应法和免疫组化法检测甲状腺组织自噬相关因子LC3B和Beclin-1的mRNA及蛋白表达。**结果:** 模型组甲状腺大量滤泡破坏, 有大量淋巴细胞浸润, 间质大量纤维组织增生; 隔药饼灸组甲状腺部分滤泡破坏, 有少量淋巴细胞浸润, 少量纤维组织增生。与正常组比较, 模型组大鼠血清TPOAb、TGAb、IL-1β、IL-6和TNF-α的浓度均升高($P<0.05$); 模型组甲状腺组织LC3B和Beclin-1的mRNA与蛋白表达水平均降低($P<0.05$)。与模型组相比, 隔药饼灸组大鼠血清TPOAb、TGAb、IL-1β、IL-6和TNF-α的浓度均降低($P<0.05$); 隔药饼灸组甲状腺组织LC3B和Beclin-1的mRNA与蛋白表达水平均升高($P<0.05$)。甲状腺组织LC3B和Beclin-1的mRNA及蛋白表达水平与血清TPOAb及TGAb的浓度均呈负相关。**结论:** 隔药饼灸能减轻EAT大鼠甲状腺的炎性反应, 降低血清TPOAb及TGAb水平。这可能与调节大鼠甲状腺组织自噬相关因子LC3B和Beclin-1的mRNA及蛋白表达有关。

**【中图分类号】**R2-03　　**【文献标志码】**A

Hashimoto thyroiditis (HT) is a common type of autoimmune thyroid disease (AITD), also known as chronic lymphocytic thyroiditis (CLT)[1]. Early stages of HT are only positive for thyroid autoantibodies of anti-thyroid peroxidase antibodies (TPOAb) and anti-thyroglobulin antibodies (TGAb), where clinical symptoms may be absent[2-4]. Epidemiological surveys have shown that the incidence of HT in China is about 1.6%[5], and it occurs mainly in middle-aged women with a male-to-female ratio of 1:4 to 1:10[6-7]. Most scholars now believe that the occurrence and development of HT are associated with genetic, environmental, and immune disorders[8]. Specific therapies for HT are lacking in clinical practice. Treatments include thyroxine replacement, antithyroid, selenium preparations, glucocorticoid, and surgery[9-11]. HT has no clear name in traditional Chinese medicine (TCM) and is classified as a "goiter disease". According to TCM, this disease is located on the bilateral sides of the front neck, mainly involving the spleen, kidney, liver, and even the heart. The pathogenesis of HT is deficiency in root cause and excess in symptoms, intermingling both deficiency and excess[12]. In clinical practice, moxibustion has been shown to regulate the expression of thyroid autoantibodies and improve thyroid function and quality of life in HT patients[13-15]. Experimental studies also found that herbal cake-partitioned moxibustion improved the histomorphological changes of thyroid glands in rats with experimental autoimmune thyroiditis (EAT), reduced the levels of thyroid autoantibodies of TPOAb and TGAb in the serum of rats, and decreased the expression levels of inflammatory factors such as interleukin-6 (IL-6), interleukin-17 (IL-17), and interleukin-23 (IL-23)[16-17]. Recent studies have found that autophagy in thyroid follicular epithelial cells is involved in the development of CLT; autophagy and autophagy-related proteins play important roles in the development and progression of autoimmune thyroid disease[18-19]. In this study, we prepared an EAT rat model by combining antigen immunization with iodine induction; investigated the anti-inflammatory effect of herbal cake-partitioned moxibustion on EAT thyroids, and the effect on the expression of thyroid autophagy-related factors, the microtubule-associated protein light chain 3 (LC3B) and Beclin-1; analyzed the correlation between the thyroid autophagy-related factors LC3B and Beclin-1 with the concentrations of thyroid autoantibodies (TPOAb and TGAb).

## 1 Materials and Methods

### 1.1 Experimental animals

Thirty-four clean-grade female Sprague-Dawley rats, body mass (100±20) g, were purchased from Shanghai SLAC Laboratory Animal Co., Ltd., China (Animal Certificate No. 201700050695) and housed in the Experimental Animal Center of Shanghai University of TCM. The rearing environment was alternate 12 h/12 h circadian rhythms with a room temperature of (20±2) ℃ and relative humidity of 40%-60%. All rats were acclimatized with standard feed and clean drinking water for 1 week before starting the experiment. The whole experimental process was performed in strict accordance with the Experimental Animal Management Regulations of Shanghai University of TCM and passed the ethical review of experimental animals from Shanghai University of TCM (Ethical Review No. PZSHUTCM2100409016).

### 1.2 Main reagents and instruments

Porcine thyroglobulin (PTg, Cat. No. A9011, Tianfu New Area, Huayang Zhenglong Biochemical Products Research Laboratory, China); complete Freund's adjuvant (CFA, Cat. No. F5881, Sigma, USA); incomplete Freund's adjuvant (IFA, Cat. No. F5506, Sigma, USA); phosphate-buffered saline (PBS, Cat. No. 806544, Sigma, USA); sodium iodide (Cat. No. V900054, Sigma, USA); moxa (Cat. No. HY-AR-2-01, Nanyang Han Medical Moxa Co., Ltd., China); rat TPOAb (Cat. No. ml003369), TGAb (Cat. No. ml003044), interleukin-1β (IL-1β, Cat. No. ml037361), interleukin-6 (IL-6, Cat. No. ml102828), and tumor necrosis factor-α (TNF-α, Cat. No. ml002859) enzyme-linked immunosorbent assay (ELISA) kits (Shanghai EnzymeLink Biotechnology Co., Ltd., China).

Low-temperature benchtop high-speed centrifuge (Thermo Scientific, USA); benchtop frozen high-speed centrifuge (Eppendorf, Germany); fully automatic dewatering machines (Leica, Germany); tissue grinder (Shanghai Jingxin Technology, China); spectrophotometer (Thermo Scientific, USA); polymerase chain reaction (PCR) instrument (Applied Biosystems, USA); real-time fluorescence quantitative PCR (RT-qPCR, Roche, Switzerland); gel imaging system (Shanghai Tennant Technology Co., Ltd., China).

### 1.3 Experimental methods[16-17]

#### 1.3.1 Experimental grouping and EAT modeling

All rats were randomly divided into a normal group (*n*=12) and a modeling group (*n*=22) after 1 week of adaptive feeding. PTg was dissolved in PBS to make a solution of 2 mg/mL and then mixed with an equal volume of CFA or IFA to fully emulsify into a white suspension for rat immunization. PTg emulsified by CFA (100 μL for each rat) was injected subcutaneously at multiple points on rat foot pads of both hindlimbs in the modeling group on day 1 of the experiment. To strengthen immunization, PTg emulsified by IFA (100 μL for each rat) was injected subcutaneously at multiple points on the foot pads and medial side of both hindlimbs, and the back of the rats in the modeling group on the 14th, 21th, and 28th days of the experiment, respectively. Rats in the normal group were injected with an equal amount of normal saline at the same sites as a control. During the modeling period, rats in the modeling group were fed with highly iodized water (500 mg/L of sodium iodide). Iodine water was ready to use and stored away from light. Rats in the normal group were given clean drinking water. At the end of modeling, 2 rats in the normal group and 2 rats in the modeling group were selected for model identification. Thyroid hematoxylin-eosin (HE) staining of the modeled rats showing follicular disruption and lymphocytic infiltration in the thyroid tissue indicated successful modeling (Figure 1).

The remaining rats with successful modeling were randomly and equally divided into a model group and a herbal cake-partitioned moxibustion group, with 10 rats in each group. Ten rats were kept in the normal group.

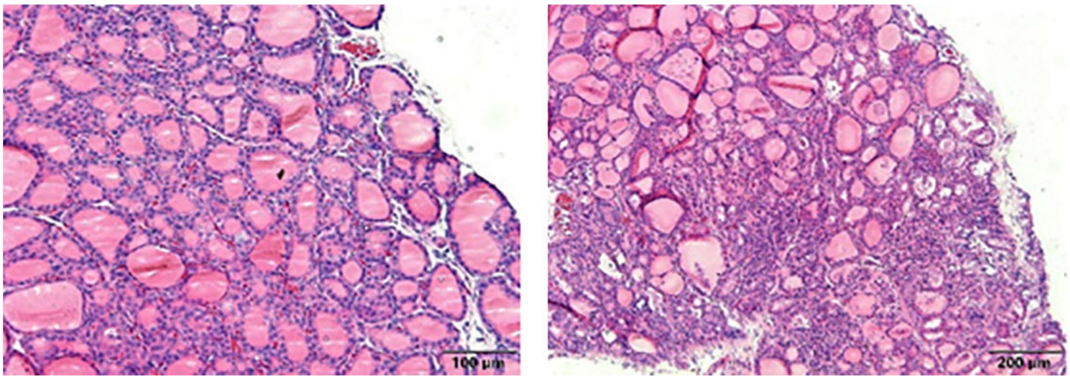

Normal rats　　Modeling rats

**Figure 1 Histopathological observation of rats' thyroid glands (hematoxylin-eosin staining, ×200)**

#### 1.3.2 Intervention methods

The intervention with herbal cake-partitioned moxibustion was performed on day 1 after the end of modeling in the herbal cake-partitioned moxibustion group. Two groups of points, Dazhui (GV14)-Mingmen (GV4) and Tiantu (CV22)-Guanyuan (CV4), were alternately treated with herbal cake-partitioned moxibustion. Rat points were positioned with reference to the *Experimental Acupuncture Science*[20]. *Zhi Fu Zi* (*Radix Aconiti Lateralis Praeparata*), *Rou Gui* (*Cortex Cinnamomi*), *Dan Shen* (*Radix Salviae Miltiorrhizae et Rhizoma*), and *Mu Xiang* (*Radix Aucklandiae*) were ground into powder and mixed with yellow wine at a certain proportion to make into herbal cakes about 0.5 cm in thickness and 1.0 cm in diameter using a mold. A moxa cone with mass about 90 mg (about 0.6 cm in bottom diameter and 0.5 cm in height) was made from the moxa wool using a mold. Rats were shaved 1 d in advance to expose the skin at the points. Rats were fixed on the rat mounting frame during the treatment, and the moxa cones were placed on the herbal cakes. Moxibustion was applied to the points mentioned above, with 2 cones (Zhuang) per point each time, once a day for 30 d. The normal and model groups did the same fixation without therapeutic intervention.

#### 1.3.3 Specimen collection

Rats in each group were fasted without water for 12 h and weighed at the end of treatment, followed by an intraperitoneal injection of 2% pentobarbital sodium [30-50 mg/(kg·bw)]. After anesthesia, 6 mL of abdominal aorta blood was collected and left to stand for 1 h, then centrifuged for 20 min at 3 600 r/min and 4 ℃. The supernatant was collected and stored in a refrigerator at -80 ℃. The left thyroid tissue was fixed with 4% paraformaldehyde, and the right thyroid tissue was stored at a -80 ℃ refrigerator.

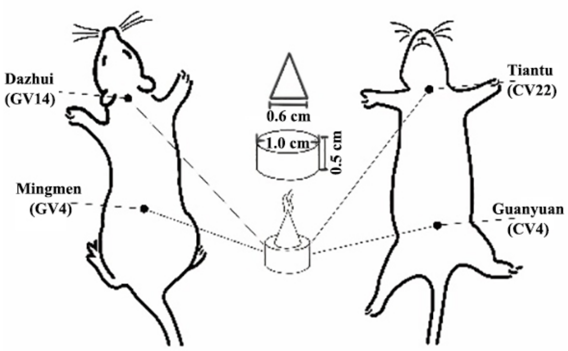

**Figure 2 Intervention schematic diagram with herbal cake-partitioned moxibustion**

1.3.4 Measurement of the serum TPOAb, TGAb, IL-6, IL-1β, and TNF-α concentrations by ELISA

The concentrations of the serum TPOAb, TGAb, IL-6, IL-1β, and TNF-α in EAT rats were measured by ELISA. The sample serum stored in the -80℃ refrigerator was thawed strictly following the ELISA kit instructions. Ten samples were measured for each group with duplicated wells for each sample, and the average value was used.

1.3.5 Histomorphological changes of thyroid observed by HE staining

The rat thyroid tissue placed in 4% paraformaldehyde fixative was dehydrated, embedded, and then cut into 4 μm wax slices. Three slices were selected from each sample. The slices were dewaxed with xylene, hydrated with gradient alcohol, stained with HE, decolorized with hydrochloric acid alcohol, dehydrated with gradient alcohol, transparent with xylene, sealed with neutral resin, and observed under a light microscope.

1.3.6 Detection of LC3B and Beclin-1 mRNA expression in thyroid tissue by RT-qPCR

The mRNA expression of LC3B and Beclin-1 in thyroid tissue was detected by RT-qPCR. The thyroid tissue frozen at -80℃ were repeatedly crushed into powder. Total RNA was extracted by Trizol lysis and reverse transcribed into cDNA for PCR amplification. Reaction conditions: pre-denaturation at 95 ℃ for 30 s, denaturation at 95 ℃ for 5 s, annealing at 53 ℃ for 30 s, extending for 30 s at 72 ℃ for 40 cycles. The reactions were performed on a LightCycler® 480 Ⅱ qPCR instrument. The data were analyzed and calculated using the instrument's own software, and the relative expression was expressed as $2^{-\Delta\Delta ct}$. The formula is as follows: ΔCt value = Ct value of the target gene - Ct value of the internal reference gene; ΔΔct value = ΔCt value - mean ΔCt value of the normal group. Then the $2^{-\Delta\Delta ct}$ operation on ΔΔct was performed. The primer sequences of each gene were designed by NCBI Primer-blast and synthesized by Jinwei Biotech Co. Ltd., Suzhou, China, as shown in Table 1.

**Table 1 Primers for mRNA detection**

| Name | Amplification length/bp | Upstream primer sequence (5'→3') | Downstream primer sequence (5'→3') |
|---|---|---|---|
| LC3B | 210 | TTCCTCCTGGTGAATGGGCA | CAGCTTCCGCTGGTAACGTC |
| Beclin-1 | 153 | AATGGAGGGGTCTAAGGCGT | GCCTGGGCTGTGGTAAGTAA |
| GAPDH | 114 | TTCAACGGCACAGTCAAGG | CTCAGCACCAGCATCACC |

Note: LC3B=Microtubule-associated protein light chain 3; GAPDH=Glyceraldehyde phosphate dehydrogenase.

1.3.7 Detection of LC3B and Beclin-1 protein expression in thyroid tissue by immunehistochemistry (IHC)

The protein expression levels of LC3B and Beclin-1 in thyroid tissue were detected by IHC. Three slices of each sample were selected, dewaxed in xylene, dewaxed in gradient alcohol to water, rinsed in PBS and boiled in EDTA antigen repair buffer for antigen repair. After PBS rinsing and incubation in 3% hydrogen peroxide solution at room temperature avoiding light, BSA block solution was added dropwise. The primary antibody against LC3B or Beclin-1 was prepared in advance at a certain ratio. Gently shook off the block solution, added primary antibody dropwise, incubated overnight at 4 ℃, rewarmed the next day, rinsed with PBS, added the secondary antibody (containing HRP marker) dropwise, rinsed again with PBS, added DAB color development solution dropwise, and put into distilled water to terminate the color development. Finally, the slices were microscopically observed, photographed, analyzed after hematoxylin re-staining, and rinsed in running water, followed by gradient alcohol dehydration, xylene transparency, and neutral gum sealing. The nuclei of thyroid follicular epithelial cells were observed microscopically as purple-blue, and the positive reaction products in the cytoplasm were yellow-brown. Three fields of view were randomly selected for each slice to determine the cumulative optical density values of positive cells, and the mean values were used for statistical analysis.

**1.4 Statistical methods**

The experimental data were statistically analyzed using the SPSS version 21.0 software. Measurement data conforming to normal distribution were expressed as mean ± standard deviation ($\bar{x}$ ±*s*), and one-way analysis of variance was used. The least significant difference method was used for the homoscedasticity data, and Games-Howell was used for the data with heterogeneity of variance. The data not conforming to a normal distribution were expressed as median (interquartile range) [M (IQR)] using a nonparametric test. Nonparametric tests were used for the enumeration data. $\alpha$=0.05 was used as the test level, and $P<0.05$ was considered a statistically significant difference.

## 2 Results

### 2.1 Effect of herbal cake-partitioned moxibustion on the histopathological changes of thyroid glands in EAT rats

The thyroid follicles in the normal group were round or oval, the follicular epithelial cells enclosing the follicles were regularly arranged, and the follicular lumen was filled with uniform glial without lymphocyte infiltration. Rats in the model group had severe destruction of thyroid follicles, significant lymphocyte infiltration, and massive interstitial fibrous tissue hyperplasia. A small amount of follicular destruction, a smaller area of lymphocyte infiltrations, and more fibrous tissue hyperplasia were seen in rats' thyroid glands of the herbal cake-partitioned moxibustion group (Figure 3).

### 2.2 Effects of herbal cake-partitioned moxibustion on the serum thyroid autoantibodies (TPOAb and TGAb) in EAT rats

Compared with the normal group, the serum TPOAb and TGAb concentrations were statistically significantly increased in rats in model group ($P<0.001$). Compared with the model group, the serum TPOAb and TGAb concentrations of rats in the herbal cake-partitioned moxibustion group were statistically significantly reduced ($P<0.001$). See Figure 4.

### 2.3 Effect of herbal cake-partitioned moxibustion on the serum protein concentrations of inflammatory factors (IL-1β, IL-6, and TNF-α) in EAT rats

Compared with the normal group, the protein concentrations of the serum IL-1β, IL-6, and TNF-α were statistically significantly increased in rats in model group ($P<0.001$). Compared with the model group, the protein concentrations of the serum IL-1β, IL-6, and TNF-α were statistically significantly reduced in the herbal cake-partitioned moxibustion group ($P<0.001$). See Figure 5.

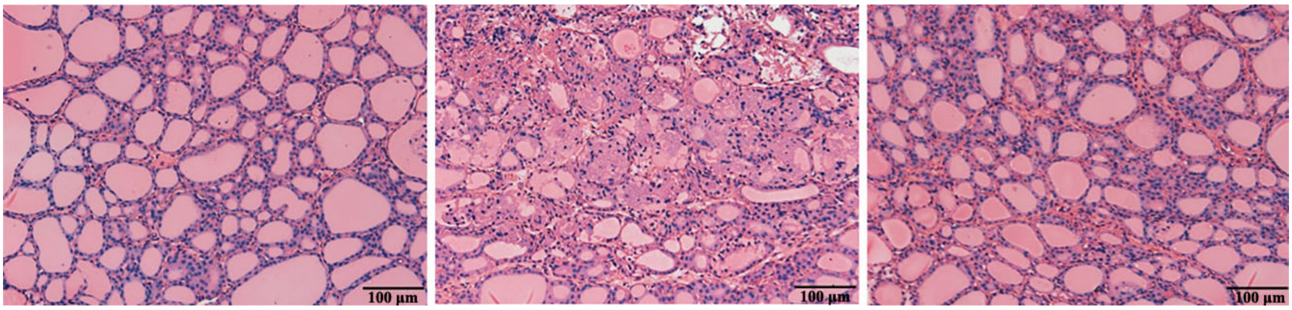

Normal group Model group Herbal cake-partitioned moxibustion group

**Figure 3 Histopathological observation of rats' thyroid glands in each group (hematoxylin-eosin staining, ×200)**

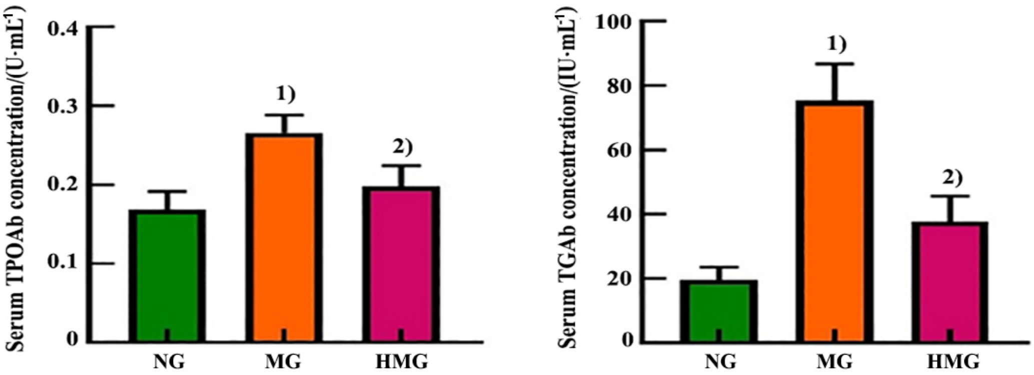

Note: NG=Normal group; MG=Model group; HMG=Herbal cake-partitioned moxibustion group; TPOAb=Thyroid peroxidase antibodies; TGAb=Thyroglobulin antibodies; compared with the normal group, 1) $P<0.001$; compared with the model group, 2) $P<0.001$.

**Figure 4 Comparison of the serum thyroid autoantibodies (TPOAb and TGAb) among groups**

Note: NG=Normal group; MG=Model group; HMG=Herbal cake-partitioned moxibustion group; IL-1β=Interleukin-1β; IL-6=Interleukin-6; TNF-α=Tumor necrosis factor-α; compared with the normal group, 1) $P$<0.001; compared with the model group, 2) $P$<0.001.

**Figure 5 Comparison of the serum inflammatory factor IL-1β, IL-6, and TNF-α concentrations among groups**

**2.4 Effects of herbal cake-partitioned moxibustion on the mRNA and protein expression levels of autophagy-related factors LC3 and Beclin-1 in the thyroid glands of EAT rats**

2.4.1 Effect of herbal cake-partitioned moxibustion on the mRNA expression levels of LC3B and Beclin-1 in the thyroid glands of EAT rats

Compared with the normal group, the mRNA expression levels of thyroid LC3B and Beclin-1 in the model group were statistically significantly lower ($P$<0.05). Compared with the model group, the mRNA expression levels of thyroid LC3B and Beclin-1 in the herbal cake-partitioned moxibustion group were statistically significantly higher ($P$<0.05). See Figure 6.

2.4.2 Effect of herbal cake-partitioned moxibustion on the protein expression of thyroid LC3B and Beclin-1 in EAT rats

Positive protein expression of the thyroid LC3B and Beclin-1 was brownish-yellow in color and expressed in the cytoplasm of thyroid follicular epithelial cells (Figure 7). Compared with the normal group, the protein expression levels of thyroid LC3B and Beclin-1 were statistically significantly lower in the model group ($P$<0.05). Compared with the model group, the protein expression levels of the thyroid LC3B and Beclin-1 in the herbal cake-partitioned moxibustion group were statistically significantly higher ($P$<0.05). See Figure 8.

**2.5 Correlation analysis of the thyroid LC3B and Beclin-1 mRNA and protein levels with the serum TPOAb and TGAb concentrations of rats in each group**

2.5.1 Correlation analysis of thyroid LC3B and Beclin-1 mRNA levels with the serum TPOAb and TGAb concentrations of rats in various groups

Six sets of experimental data of rats in each group were randomly selected for correlation analysis of the serum TPOAb and TGAb with the thyroid LC3B and Beclin-1 mRNA expression levels, which showed that the LC3B mRNA expression level was negatively correlated with concentrations of TPOAb ($P$<0.05) and TGAb ($P$<0.05). The Beclin-1 mRNA expression level was also negatively correlated with concentrations of TPOAb ($P$<0.05) and TGAb ($P$<0.05). See Figure 9.

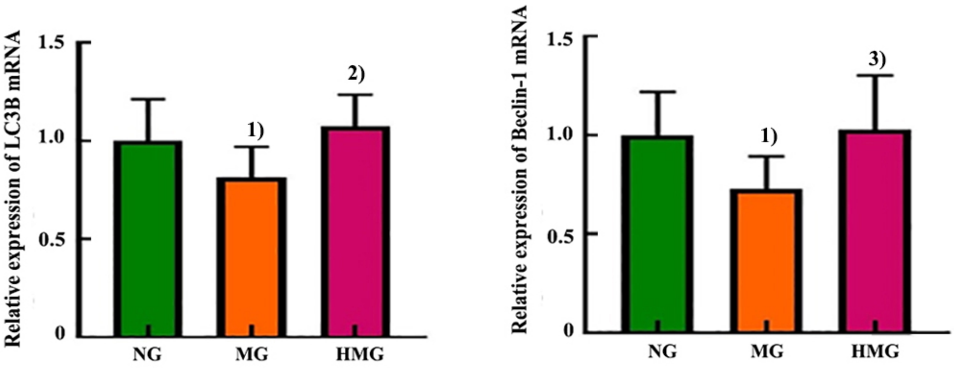

Note: NG=Normal group; MG=Model group; HMG=Herbal cake-partitioned moxibustion group; compared with the normal group, 1) $P$<0.05; compared with the model group, 2) $P$<0.01, 3) $P$<0.05.

**Figure 6 Comparison of the mRNA expression levels of the autophagy-related factors LC3B and Beclin-1 in thyroid glands among groups**

| © The author(s) for open access article 2022

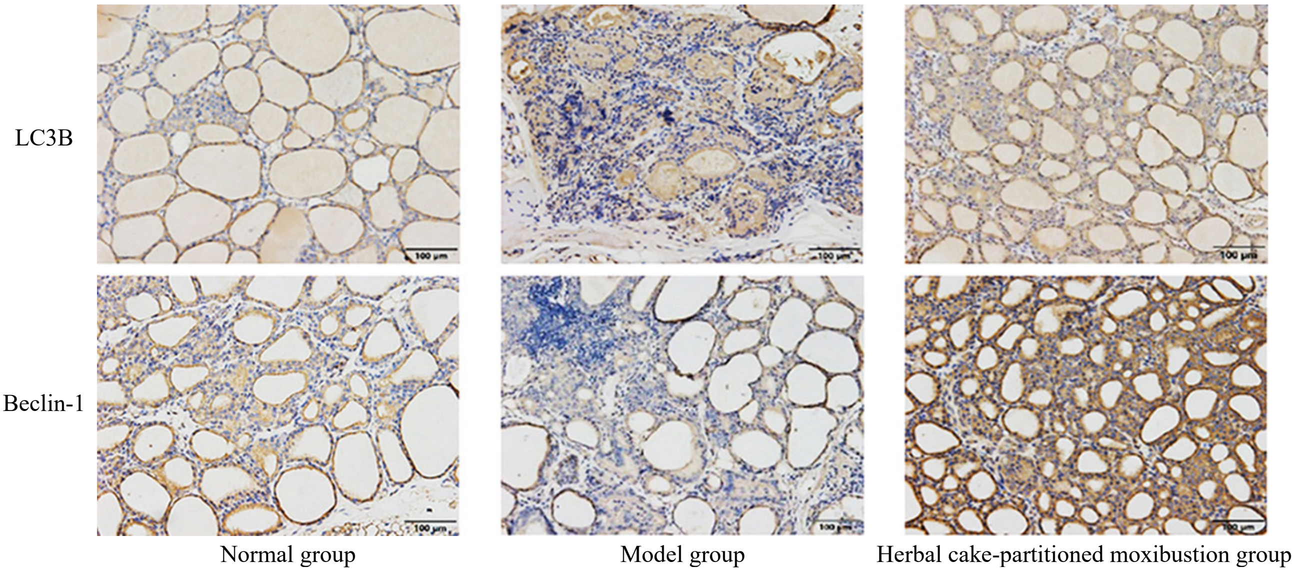

**Figure 7 Protein expression of the thyroid autophagy-related factors LC3B and Beclin-1 (immunohistochemistry, ×200)**

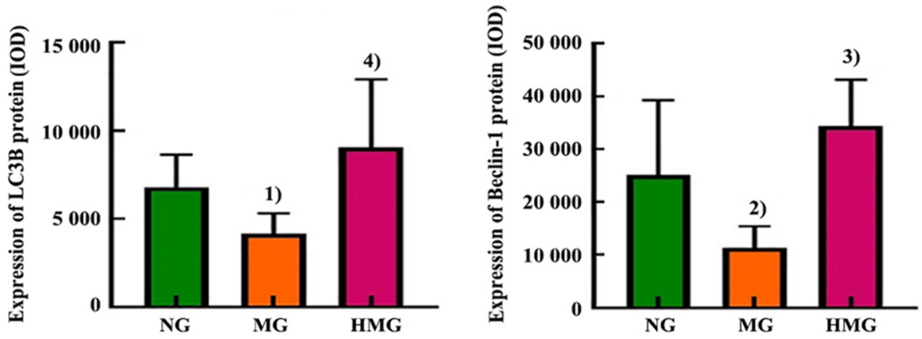

Note: IOD=Integrated optical density; NG=Normal group; MG=Model group; HMG=Herbal cake-partitioned moxibustion group; compared with the normal group, 1) $P$<0.01, 2) $P$<0.05; compared with the model group, 3) $P$<0.001, 4) $P$<0.05.

**Figure 8 Expression levels of the autophagy-related factors LC3B and Beclin-1 in thyroid glands**

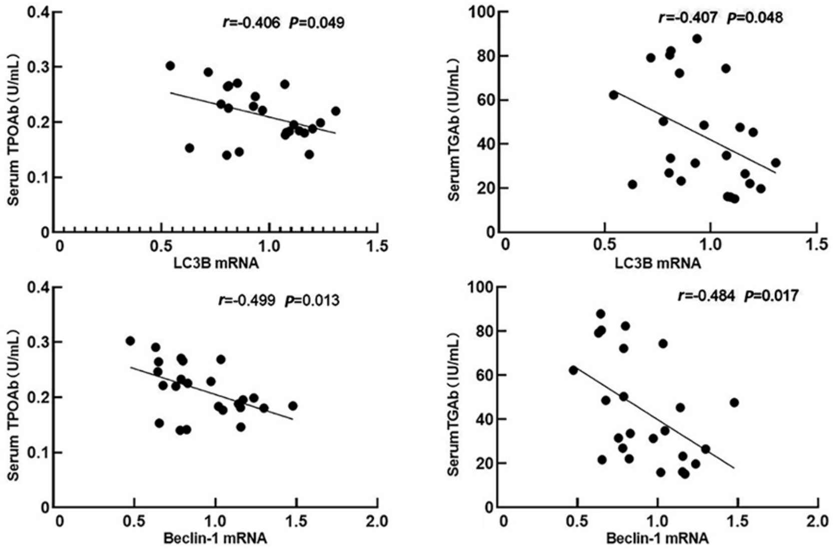

**Figure 9 Correlation analysis of the thyroid LC3B and Beclin-1 mRNAs expression with the serum TPOAb and TGAb levels**

2.5.2 Correlation analysis of the thyroid LC3B and Beclin-1 protein expression levels with the serum TPOAb and TGAb concentrations of rats in each group

Six sets of experimental data of rats in each group were randomly selected, and the correlation analysis of the thyroid LC3B and Beclin-1 protein expression with the serum TPOAb and TGAb concentrations was performed, which showed that the LC3B protein expression level was negatively correlated with concentrations of TPOAb (*P*<0.05) and TGAb (*P*<0.05). The Beclin-1 protein expression level was negatively correlated with the expression levels of TPOAb (*P*<0.05) and TGAb (*P*<0.01). See Figure 10.

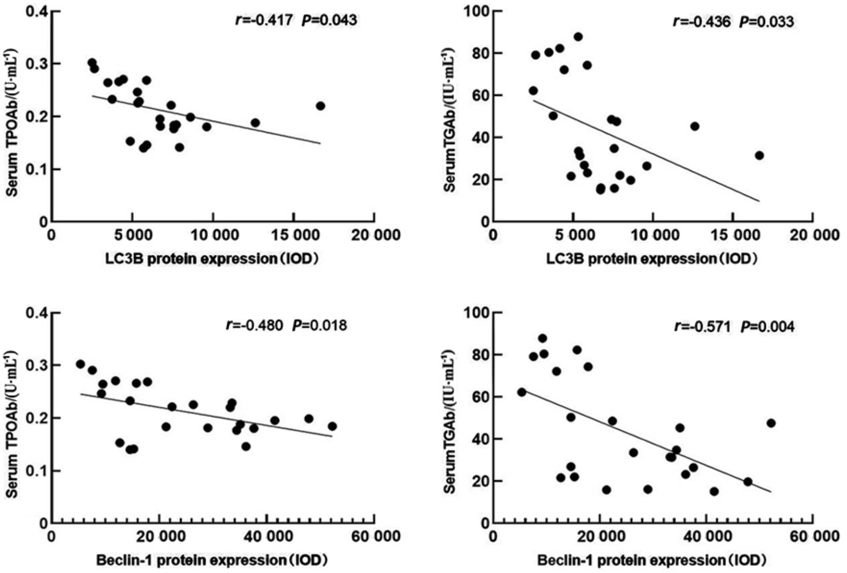

Note: IOD=Integrated optical density.

**Figure 10 Correlation analysis of thyroid LC3B and Beclin-1 protein expression with serum TPOAb and TGAb levels**

## 3 Discussion

HT does not have a clear disease name in TCM. It is classified in TCM as a "goiter disease" along with simple goiter, hyperthyroidism, and nodular goiter due to its characteristic of an enlarged thyroid gland. The pathological site of HT is on the two sides of the front neck. HT mainly affects the liver, spleen, kidney, and also heart. The mechanism of HT is the dysfunction of Zang-Fu organs, leading to Qi stagnation with phlegm retention obstructing in the front of the neck, causing stasis retention in blood vessels, and insufficiency of Qi and Yin over time, and the combination of Qi, phlegm, and stasis[12]. There is no uniform pattern differentiation of HT, but the pathogenesis is deficiency in root cause and excess in symptoms, mainly the deficiency of the spleen and kidney, and the treatment should be to reinforce the spleen and benefit the kidney[21]. The herbal cake-partitioned moxibustion has a better effect on HT with spleen and kidney Yang deficiency pattern through the warming effect of moxibustion and herbal cake. In this study, based on clinical practice and preliminary basic experimental research, two groups of points, Dazhui (GV14)-Mingmen (GV4) and Tiantu (CV22)-Guanyuan (CV4), were selected for alternate treatment with herbal cake-partitioned moxibustion. Dazhui (GV14), the crossing point of the three Yang meridians of hand and foot with the Governor Vessel, regulates all Yang Qi and plays the role of warming and tonifying Yang Qi together with the warming effect of herbal cake-partitioned moxibustion. *Lei Jing Tu Yi* (*Pictorial Appendices to the Classified Classic*) mentions that 100 cones (Zhuang) of moxibustion at Dazhui (GV14) can cure neck goiter. Mingmen (CV4) is an important point for warming the kidney and strengthening Yang, and it can enhance the anti-pathogenic Qi after moxibustion. Tiantu (CV22) is a localized point. *Lei Jing Tu Yi* records that moxibustion at Tiantu (CV22) can circulate blood and resolve goiter, and treat all initial goiter. Guanyuan (CV4), the point where the body's Yuan-Primordial Qi is hidden, reinforces the Yuan-Primordial Qi to strengthen the body after moxibustion, warms the kidney, and strengthens Yang together with moxibustion at Mingmen (CV4). XU H F[22] found in a clinical study that herbal cake-partitioned moxibustion at Dazhui (GV14) and Mingmen (CV4) was more effective in improving thyroid function compared with Western medicine treatment alone. XIA Y, *et al*[15] and WANG X Y[23] found that alternate herbal cake-partitioned moxibustion at Dazhui (GV14), Mingmen (GV4), and Guanyuan (CV4) combined with oral euthyrox could better alleviate patients' symptoms of HT. In a previous clinical study, our research team found that herbal cake-partitioned

| © The author(s) for open access article 2022

moxibustion at Dazhui (GV14), Mingmen (GV4), Tiantu (CV22), and Guanyuan (CV4) was effective in treating HT[24]. Experimental studies confirmed that herbal cake-partitioned moxibustion at Dazhui (GV14)-Mingmen (GV4) and Tiantu (CV22)-Guanyuan (CV4) effectively improved the histopathological changes of thyroid glands and reduced the serum thyroid autoimmune antibody levels in EAT rats[16-17].

In cases of diffuse goiter, a positive serum test for TPOAb and TGAb is clinically diagnostic of HT irrespective of whether the thyroid function is altered[1]. In this study, histopathological observations revealed that the number of follicles was reduced, and the area of lymphocyte infiltration was enlarged in the rat's thyroid tissue of the model group compared with the normal group. Compared with the model group, the number of follicular destruction and the area of lymphocyte infiltration in the thyroid glands of the herbal cake-partitioned moxibustion group were improved, suggesting that herbal cake-partitioned moxibustion can improve the pathological changes in the thyroid glands. TPOAb and TGAb are the hallmark antibodies to HT[25-26]. Serum TPOAb and TGAb level detection in clinical practice is of great importance to improve the accuracy, specificity, and sensitivity of HT diagnosis[27-28]. The results of this study showed that the serum TPOAb and TGAb concentrations of rats in the model group were significantly higher than those in the normal group. The serum TPOAb and TGAb concentrations in the herbal cake-partitioned moxibustion group were significantly lower than those in the model group, suggesting that the herbal cake-partitioned moxibustion reduces the levels of thyroid autoantibodies, thus to control the disease development. Herbal cake-partitioned moxibustion effectively improved the histopathological changes of thyroid glands in EAT rats, reduced the levels of serum thyroid autoantibodies (TPOAb and TGAb), lowered the expression levels of serum inflammatory factors (IL-17 and IL-23), reduced inflammatory responses, and played a role in protecting thyroid glands[16]. Clinical studies have found that the inflammatory response in HT patients is associated with inflammatory factors such as IL-1β, IL-6, and TNF-α[29-30]. Animal studies have found that the drug osteopontin has a therapeutic effect on EAT rats by reducing serum levels of cytokines such as TNF-α, IL-1, and IL-6[31]. The results of this study showed that the concentrations of serum inflammatory factors (IL-1β, IL-6, and TNF-α) were significantly higher in the model group compared with the normal group. The concentrations of serum inflammatory factors (IL-1β, IL-6, and TNF-α) in EAT rats were significantly reduced by herbal cake-partitioned moxibustion, suggesting that herbal cake-partitioned moxibustion can reduce the inflammatory response and inhibit the release of inflammatory factors in thyroid glands of EAT rats. A previous clinical study by our research team found that the herbal cake-partitioned moxibustion was effective in reducing serum inflammatory factors [interleukin-2 (IL-2), interleukin-4 (IL-4), and interferon-γ (IFN-γ)] levels in patients with HT[32]. Relevant animal experimental studies have found that herbal cake-partitioned moxibustion can effectively reduce serum levels of inflammatory factors such as IL-6, IL-17, IL-23, and TNF-α in rats[16-17]. In conclusion, combining the results of thyroid histomorphology, thyroid autoantibodies, and inflammatory factors in serum, it was found that herbal cake-partitioned moxibustion could mitigate the destruction of thyroid tissue follicles and decrease the exudation of lymphocytes and plasmocytes in EAT rats, producing a better protective effect on thyroid glands of EAT rats.

Based on the confirmed protective effect of herbal cake-partitioned moxibustion on the thyroid glands of EAT rats, here we explored the immunomodulatory mechanism from the perspective of cellular autophagy, which plays an important role in autoimmune diseases[33-37]. Recent studies have found that cellular autophagy is closely related to the occurrence and development of AITD[18]. LI B N, *et al*[19] analyzed the isolated human thyroid follicular cells by flow cytometric analysis and concluded that patients with HT had low levels of tissue autophagy, and thyroid follicular cell autophagy was involved in the development of HT. microtubule-associated protein light chain 3 (LC3) is a key marker protein for autophagy[38-39]. LC3B/LC3-Ⅱ detection is for the LC3 assay[39-40]. XU C, *et al*[41] found that autophagy-related protein LC3-Ⅱ was reduced in thyroid tissue of HT patients. YANG Q J, *et al*[42] found that the autophagy level was reduced and the expression of autophagy-related protein LC3B was inhibited in thyroid epithelial cells of HT patients. Beclin-1 is a mammalian autophagy gene inhibiting tumorigenesis and also a key factor in autophagy activation, playing an important role in the initiation phase of autophagy[43]. In this experiment, the level of cellular autophagy was measured by detecting the expression of LC3B and Beclin-1. The mRNA and protein expression levels of LC3B and Beclin-1 in thyroid tissue of EAT rats in the model group were lower than those in the normal group, and herbal cake-partitioned moxibustion increased the mRNA and protein expression levels of LC3B and Beclin-1 in thyroid tissue of EAT rats. It is now believed that, within the normal activation range, autophagy plays a protective role for cells by removing defective proteins and damaged organelles, and invading pathogens that cause various diseases; that is, cellular autophagy helps to suppress inflammatory reactions[44-45]. Several studies have shown that the increased cellular autophagy increases the expression of LC3 and Beclin-1[18-19]. The expression of autophagy-related factors LC3 and Beclin-1 in thyroid follicular cells of HT patients is low, and promoting autophagy effectively reduces the expression of inflammatory factors such as IL-1β, IL-6, and TNF-α in serum and attenuates inflammatory responses[46-49]. In addition, correlation analysis showed that the mRNAs and proteins of autophagy-related factors (LC3B and Beclin-1) in the thyroid tissue were negatively correlated with thyroid autoantibodies of TPOAb and TGAb in the serum, respectively. The results suggest that herbal cake-partitioned moxibustion may protect thyroid glands by enhancing the level of thyroid autophagy in EAT rats, decreasing the concentrations of serum inflammatory factors, and reducing the inflammatory response of thyroid tissue.

Based on the clinical and experimental studies on herbal cake-partitioned moxibustion on HT patients and EAT rats, this experiment initially confirmed the effect of herbal cake-partitioned moxibustion on EAT rats in reducing the levels of thyroid autoantibodies and inflammatory symptoms and explored the effect of herbal cake-partitioned moxibustion on autophagy-related factors in the thyroid tissue of EAT rats. At present, only an autophagy-regulated phenomenon has been observed, and we will further elucidate how autophagy is regulated by herbal cake-partitioned moxibustion using the inhibitors and knockout mice to provide more exact laboratory information and scientific basis for the promotion and application of clinical herbal cake-partitioned moxibustion for HT.

**Conflict of Interest**

The authors declare that there is no potential conflict of interest in this article.

**Acknowledgments**

This work was supported by the National TCM Leading Talents Support Program-Qihuang Scholars (国家中医药领军人才支持计划-岐黄学者); National Program on Key Basic Research Project “973 Program” (国家重点基础研究发展计划“973 计划项目”, No. 2015CB554501); Projects of National Natural Science Foundation of China (国家自然科学基金项目, No. 82074551, No. 81704176); Youth Project from Department of Science and Education, Shanghai Health Commission (上海市卫健委科教处青年基金, No. 20194Y0013).

**Statement of Human and Animal Rights**

The treatment of animals conformed to the ethical criteria in this experiment.

Received: 2 March 2022/Accepted: 16 June 2022
